# Structural and functional fetal cardiac imaging using low field (0.55 T) MRI

**DOI:** 10.3389/fped.2024.1418645

**Published:** 2024-09-03

**Authors:** Charlie Yuli Zhang, Michela Cleri, Tomas Woodgate, Paula Ramirez Gilliland, Simi Bansal, Jordina Aviles Verdera, Alena U. Uus, Vanessa Kyriakopoulou, Kamilah St Clair, Lisa Story, Megan Hall, Kuberan Pushparajah, Joseph V. Hajnal, David Lloyd, Mary A. Rutherford, Jana Hutter, Kelly Payette

**Affiliations:** ^1^ Research Department of Early Life Imaging, School of Biomedical Engineering and Imaging Sciences, King’s College London, London, United Kingdom; ^2^ Biomedical Engineering Department, School of Biomedical Engineering and Imaging Sciences, King’s College London, London, United Kingdom; ^3^ London Collaborative Ultra High Field Systems (LoCUS), King’s College London, London, United Kingdom; ^4^ Department of Congenital Heart Disease, Evelina Children Hospital, London, United Kingdom; ^5^ Department of Women & Children’s Health, King’s College London, London, United Kingdom; ^6^ Smart Imaging Lab, Radiological Institute, University Hospital Erlangen, Erlangen, Germany

**Keywords:** low-field, fetal cardiac, magnetic resonance imaging, blood flow measurements, congenital heart disease, fetal imaging

## Abstract

**Purpose:**

This study aims to investigate the feasibility of using a commercially available clinical 0.55 T MRI scanner for comprehensive structural and functional fetal cardiac imaging.

**Methods:**

Balanced steady-state free precession (bSSFP) and phase contrast (PC) sequences were optimized by *in utero* studies consisting of 14 subjects for bSSFP optimization and 9 subjects for PC optimization. The signal-to-noise ratio (SNR) of the optimized sequences were investigated. Flow measurements were performed in three vessels, umbilical vein (UV), descending aorta (DAo), and superior vena cava (SVC) using the PC sequences and retrospective gating. The optimized bSSFP, PC and half-Fourier single shot turbo spin-echo (HASTE) sequences were acquired in a cohort of 21 late gestation-age fetuses (>36 weeks) to demonstrate the feasibility of a fetal cardiac exam at 0.55 T. The HASTE stacks were reconstructed to create an isotropic reconstruction of the fetal thorax, followed by automatic great vessel segmentations. The intra-abdominal UV blood flow measurements acquired with MRI were compared to ultrasound UV free-loop flow measurements.

**Results:**

Using the parameters from 1.5 T as a starting point, the bSSFP sequences were optimized at 0.55 T, resulting in a 1.6-fold SNR increase and improved image contrast compared to starting parameters, as well as good visibility of most cardiac structures as rated by two experienced fetal cardiologists. The PC sequence resulted in increased SNR and reduced scan time, subsequent retrospective gating enabled successful blood flow measurements. The reconstructions and automatic great vessel segmentations showed good quality, with 18/21 segmentations requiring no or minor refinements. Blood flow measurements were within the expected range. A comparison of the UV measurements performed with ultrasound and MRI showed agreement between the two sets of measurements, with better correlation observed at lower flows.

**Conclusion:**

We demonstrated the feasibility of low-field (0.55 T) MRI for fetal cardiac imaging. The reduced SNR at low field strength can be effectively compensated for by strategically optimizing sequence parameters. Major fetal cardiac structures and vessels were consistently visualized, and flow measurements were successfully obtained. The late gestation study demonstrated the robustness and reproducibility at low field strength. MRI performed at 0.55 T is a viable option for fetal cardiac examination.

## 1 Introduction

Congenital heart disease (CHD) is a leading cause of infant morbidity and mortality, with an incidence of around 8/1,000 live births (1, 2). Early and accurate diagnosis of CHD provides opportunities for prenatal planning, counselling and life-saving treatment to be delivered immediately after birth, improving postnatal outcomes (3). Fetal echocardiography remains the gold standard for prenatal diagnosis due to its ease of use, speed, and diagnostic accuracy (4–6). However, MRI has gained popularity as a complementary diagnostic tool to echocardiogram for the evaluation of fetal cardiac structures and treatment planning for fetuses with CHD due to its excellent soft tissue contrast, larger field of view (FOV), and improved visualization of the fetal anatomy (7). In addition to providing morphological information, MRI can be used to investigate and quantify fetal heart function using flow measurements (8). Nonetheless, fetal cardiac MRI is challenging due to the small size of fetal cardiac structures, the short fetal cardiac cycle of 330–540 ms (7), motion from maternal breathing and gross fetal movement, and the lack of synchronization of the fetal heartbeat and image acquisition due to the absence of a fetal electrocardiogram (ECG) signal (7, 9, 10).

To address these challenges in structural fetal cardiac imaging, fast single-shot imaging sequences such as the half-Fourier single shot turbo spin-echo (HASTE) and the balanced steady-state free precession (bSSFP) sequence have been employed to overcome motion corruption and acquire static 2D slices (10, 11). However, these generate highly anisotropic images with in-plane resolutions generally ranging from 0.5–1 mm and slice thickness of 3–5 mm, which limits evaluation of the fetal cardiac anatomy (7, 9). 3D evaluation using these sequences has been made possible through the development of methods such as deformable slice-to-volume registration (dSVR) (12–14). dSVR accurately reconstructs multiple 2D images into a 3D space while employing motion correction (12) creating a higher-resolution isotropic volume for accurate structural evaluation (14).

In order to perform blood flow measurements in the major fetal vessels, phase contrast (PC) sequences have been used. In these sequences, synchronization with the fetal heart rate and cardiac cycle is essential, particularly for arterial blood flow. Retrospective gating methods such as metric optimized gating (MOG) and an MR compatible Doppler ultrasound device have been utilized for this purpose (15, 16). MOG is an image-based retrospective gating algorithm. It enforces data consistency using an entropy-based metric to reorganize the acquired data into a complete cardiac cycle, thereby gating and estimating the heart rate without an ECG signal (15). The use of a Doppler ultrasound device for cardiac synchronization has been shown to be effective for fetal cardiac applications, including both structural cine imaging and blood flow measurements (17–19).

Fetal cardiac MRI is usually performed at conventional field strengths of 1.5 T and 3.0 T (19–22). However, increasing the field strength, while bringing the benefit of higher signal-to-noise ratio (SNR), also carries challenges, some of which are amplified in fetal MRI. $B_{0}$ field inhomogeneities result in geometric distortion artifacts in images, especially next to tissue-air interfaces, such as when gas is present in the maternal bowels next to the uterus. Similarly, increased $B_{1}$ field inhomogeneity leads to bias field and inconsistent image appearance, impacting fetal MRI images (23). Higher field strengths also result in higher specific absorption rate (SAR) values, which is a limiting factor for fetal cardiac MRI, which typically relies on SAR-intense sequences such as HASTEs and bSSFPs. Addressing these challenges at 1.5 T or 3 T requires advanced tools such as image-based shimming or bias field correction in the post-processing (24, 25). Furthermore, commonly used bSSFP techniques for cardiac MRI are affected by wave-length dependent banding artifacts and require careful optimization (26). These challenges, in conjunction with the recent availability of clinical low field (0.55 T) MRI scanners have led to a revival of low field MRI, which presents an exciting opportunity for fetal cardiac MRI.

At 0.55 T, the reduced magnetic susceptibility reduces distortion artifacts and thus eliminates the need for advanced shimming techniques. The longer T2* allows for longer signal readouts, enabling a longer echo time (TE) and narrower bandwidth (BW), and the shorter T1 times allows for shorter repetition time (TR) settings (27). The larger bore size (80 cm) available with current clinical 0.55 T scanners, provides wider access for obese and claustrophobic patients, as well as an overall more comfortable imaging experience for pregnant individuals (28), particularly in late gestation. The lower SAR values at a lower field strength, allows for higher flip angles (FA) to be used while maintaining safety levels, improving image contrast. However, the decrease in SNR which is proportional to the strength of the main magnetic field $B_{0}$ remains the main challenge when moving to low field.

Despite the loss in SNR at lower field strengths, studies have shown the feasibility of both structural and functional fetal MRI at 0.55 T (29, 30). Adult cardiac MRI has also been demonstrated and optimized at 0.55 T for various applications (31–34), however, fetal cardiac MRI remains largely unexplored. The bSSFP sequence is of particular interest for fetal cardiac imaging at low field, the inherent T2/T1 contrast is enhanced at low field, and the properties of low field allow for lower BW and higher FA settings to improve image quality (30). Preliminary results show the feasibility of using the bSSFP sequence for fetal imaging at 0.55 T (30, 35). However, SNR quantification to measure the SNR gained through sequence optimization at 0.55 T is lacking. Effective planning of PC sequences for blood flow measurements requires high quality anatomical images at low field, and subsequent fetal blood flow measurements using PC data have not been attempted at low field strengths. Lastly, the capabilities of various fetal cardiac imaging techniques to visualize different cardiac structures at low field remains to be assessed.

**Figure 1:**
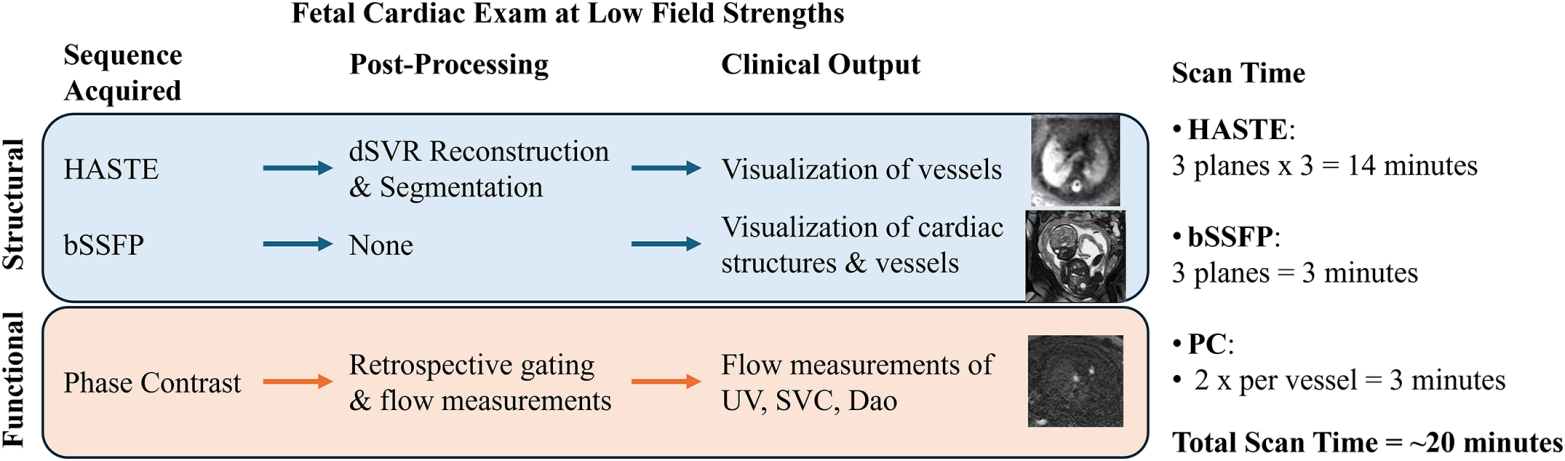
Overview of the fetal cardiac exam at low field strengths. HASTE is acquired in three planes with three stacks per plane in order to perform the reconstruction, bSSFP is acquired once per plane (for visualization and for PC planning), and PC is acquired twice for each of the three vessels (HASTE, half-Fourier single-shot turbo spin-echo; bSSFP, balanced steady-state free precession; SAR, specific absorption rate; dSVR, deformable slice-to-volume reconstruction; UV, umbilical vein; SVC, superior vena cava; DAo, descending aorta).

Here we present a comprehensive morphological and functional cardiac examination optimized on a 0.55 T commercial MRI scanner (Figure 1). We first optimize the bSSFP and PC sequences and demonstrate the ability to visualize the major vessels and measure blood flow at 0.55 T in three vessels. We provide image quality evaluation of the optimized sequences in order to assess its diagnostic quality. The robustness and reproducibility of our protocol is investigated by prospectively imaging a cohort of late gestation fetuses at low field strengths, which would be especially suited for low field imaging due to the larger bore size and lower T2* values present in late gestation. Finally, the umbilical vein (UV) blood flow measurements obtained from MRI are compared to UV measurements obtained with ultrasound, the current clinical gold standard for *in utero* flow measurements. Through this study, we demonstrate the feasibility of using a commercially available 0.55 T MRI scanner for performing a comprehensive fetal cardiac MR imaging examination consisting of both structural and functional imaging.

## 2 Materials and methods

### 2.1 MR acquisition

All scans were performed using a clinical 0.55 T scanner (MAGNETOM Free.Max, Siemens Healthcare, Erlangen, Germany), using a 9-element spine coil integrated into the patient table alongside a 6-element flexible coil (BioMatrix Contour Coil, Siemens Healthcare, Erlangen, Germany). *In utero* Fetal MRI was acquired as part of three ethically approved prospective single-center studies (REC 21/LO/0742, REC 22/YH/0210, REC 23/LO/0685) performed between June 2023 and February 2024 at St Thomas’ Hospital in London, UK, a tertiary referral center. Participants were recruited prospectively, with inclusion criteria of a singleton pregnancy and maternal age over 18 years. Exclusion criteria were multiple pregnancies, maternal age < 18 years, lack of ability to consent, weight > 200 kg, and contraindications for MRI such as MR unsafe implants and extreme claustrophobia. Participants were scanned with continuous heart rate and saturation monitoring, and intermittent blood pressures in the head-first supine position with frequent verbal interaction. The data is available from the corresponding author upon reasonable academic request (REC 21/LO/0742).

### 2.2 bSSFP sequence optimization

#### 2.2.1 Sequence optimization

**Figure 2:**
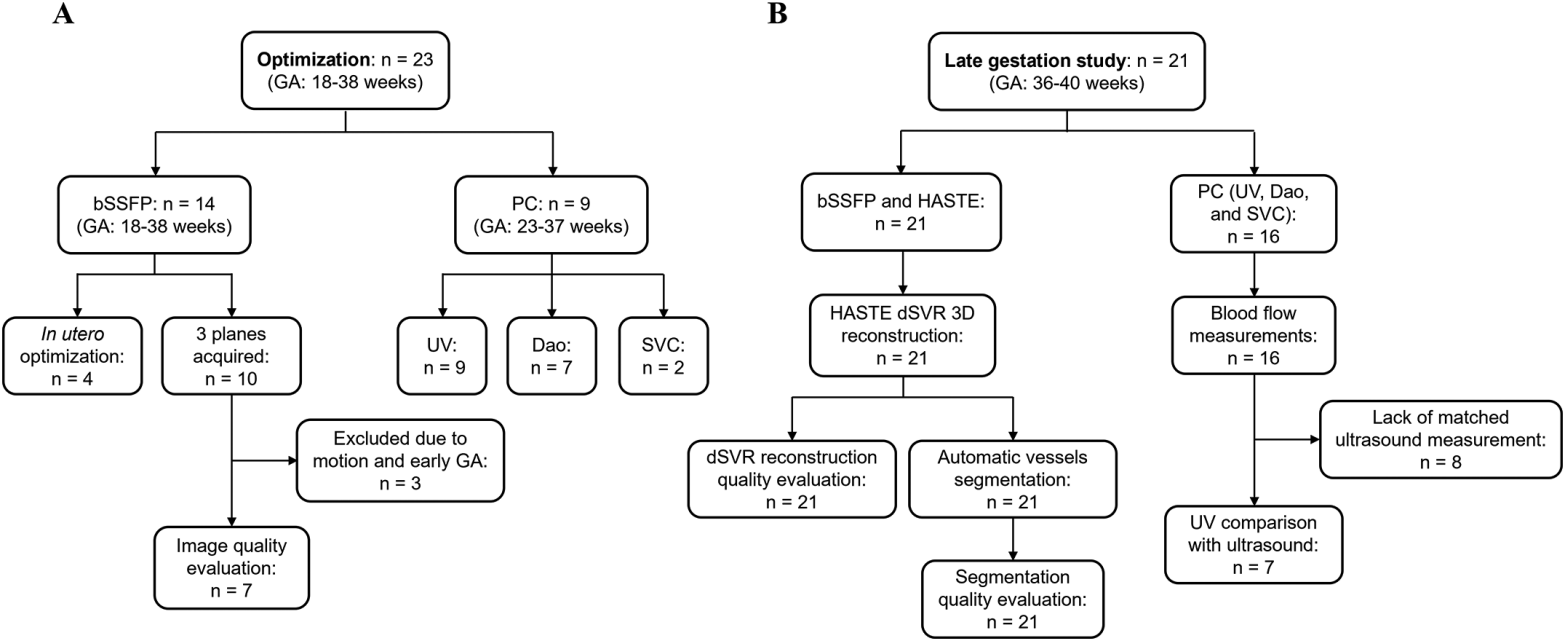
Flow chart of included subjects. **(A)** Optimization cohort used to optimize bSSFP and PC sequences. **(B)** Late gestation study cohort to investigate using the optimized sequences for a comprehensive structural and functional fetal cardiac protocol. *n*, number of subjects scanned.

Current clinical bSSFP sequence parameters from a 1.5 T scanner were used as a starting point (Table 1). Parameters were initially optimized in phantom experiments (Supplementary Material), followed by *in utero* optimization. The *in utero* study consists of 14 subjects (Figure 2A). Coronal bSSFP stacks were first acquired in 4 subjects for parameter optimization, and the optimized parameters were then used to acquire bSSFP stacks in all three planes for 10 subjects. The sequence optimization focused on varying the FA and BW to increase SNR, as these two parameters heavily contribute to the SAR and image artifacts at higher field strengths. Meanwhile, the TE and TR were set to the minimum values. Parameters that resulted in the highest SNR while maintaining relatively high resolution and acquisition time were determined to be optimal. The range of tested parameters can be found in Table 1. The optimized parameters as determined by the phantom experiments and subsequent *in utero* study were used [TR/TE = 649.2/4.09 ms, BW = 250 Hz/Px, FA = 120°, FOV = 350 × 350 mm^2^, acquired matrix size = 148 × 272, interpolation on, reconstructed resolution = 0.7 × 0.7 × 4.5 mm^3^, phase encoding lines = 102, asymmetric echo off, partial Fourier = 6/8, and acquisition time = 42 s (Table 1)]. The *in utero* SNR was measured as the apparent SNR, defined as the mean signal in a region of interest (ROI) divided by the standard deviation of a background region (36). To assess SNR variations across different fetal anatomical regions, multiple ROIs were defined, including brain white matter, lung, heart, placenta, and amniotic fluid. The apparent noise was measured in three separate ROIs in the background region, the mean was then calculated to ensure more precise noise measurement.

**Table 1:** bSSFP and PC sequence parameters.

Sequence	TR (ms)		TE (ms)		BW (Hz/px)	FA (°)	FOV (mm^2^)	In-plane resolution (mm^2^)	Slice thickness (mm)	GRAPPA	Acquisition time (s)
bSSFP (1.5 T)	569.4		3.11		514	88	350 × 350	0.7 × 0.7	5.0	2	20 (1.5 T), 25 (0.55 T)
bSSFP (Test)	476.9–695.7		2.99–4.38		250, 514	88, 120	350 × 350	0.7 × 0.7, 1.0 × 1.0, 1.25 × 1.25	3.5, 4.0, 4.5, 5.0	2	25–50
bSSFP (Optimized)	649.2		4.09		250	120	350 × 350	0.7 × 0.7	4.0 or 4.5	2	42
	TR (ms)	TE (ms)	BW (Hz/px)	FA (deg)	FOV (mm^2^)	Resolution (mm^2^)	Slice Thickness (mm)	GRAPPA	Segments	Phase-Oversampling (%)	Acquisition Time (s)
PC (1.5 T)	51.4	4.00	449	20	240 × 240	1.3 × 1.3	5.0	0	4	50	20
PC (Test)	53.16–98.30	4.89–5.31	220–449	20–40	240 × 240–300 × 300	1.3 × 1.3–1.4 × 1.4	8.0, 5.0	0–3	3–5	50–100	20–60
PC (Optimized)^a^	98.3, 78.6	5.31	220	40	300 × 300	1.4 × 1.4	5.0	2, 3	5, 4	50	20

Clinical sequence parameters at 1.5 T were used as a starting point, combinations of different parameters were tested *in utero*. The optimized parameters were decided based on SNR, contrast, and image resolution.

^a^ Two sets of optimized PC parameters used: TR = 98.3 ms, GRAPPA = 2, segments = 5, and TR = 78.6 ms, GRAPPA =3, segments = 4. Resolution values are displayed with no interpolation.

#### 2.2.2 bSSFP: image quality evaluation

Two experienced fetal cardiologists (DL, TW) performed an image quality evaluation for 7 bSSFP cases acquired with the optimized parameters in all three orthogonal planes (Figure 2A). Images were scored based on the visibility of 12 different cardiac structures, according to the methodology used by Geiger et al. (37). The structures examined included the cardiac position, the right and left ventricle (RV, LV), the right and left atrium (RA, LA), superior and inferior vena cava (SVC, IVC), left and right ventricular outflow track (LVOT, RVOT), aortic arch, descending aorta (DAo), and ductal arch. For each structure, a score of 0 (not visible) to 3 (excellent visibility) was assigned. For the visualization assessment, the percentage of visible structures was calculated, where scores of 0 and 1 were regarded as not visible, and scores of 2 and 3 were regarded as visible. The average values for each structure were calculated.

### 2.3 Phase contrast sequence optimization and blood flow measurement

bSSFP sequences with the optimized parameters (Table 1) from the previous experiments were acquired in three orthogonal planes for planning of the PC sequences. Initial 2D PC sequence parameters were translated from the existing 1.5 T sequence (which has been set up to use a Doppler ultrasound device for gating) and resulted with the following parameters on 0.55T: FA = 20°, BW = 449 Hz/Px, segments = 4, acquired matrix size = 208 × 208, reconstructed resolution = 1.3 × 1.3 × 5.0 mm^3^, no acceleration (i.e., GRAPPA), phase encoding lines = 208, asymmetric echo strong, partial Fourier off, and acquisition time = 60 s (Table 1). The original 1.5 T PC sequence can also be found in Table 1. The PC sequence was optimized in 9 subjects (Figure 2A), where the parameters outlined in Table 1 were explored, with the goal of a scan time less than 20 s to limit the impact of fetal motion while achieving higher SNR. The SNR was calculated in the magnitude image before retrospective gating using the same approach as Section 2.2.1 and was compared across the varying scan parameters. Three vessels were imaged using the PC sequences and the velocity encoding (venc) was set according to the vessel: UV: venc = 50 cm/s, DAo: venc = 150 cm/s, and SVC: venc = 100 cm/s. Each acquired sequence underwent a visual assessment for motion, and any scans deemed to have too much motion were discarded (38). PC sequences were retrospectively gated using MOG (15), and mean flow measurements were carried out using cvi42 V5.11 (Circle Cardiovascular Imaging Inc. Calgary, Canada).

### 2.4 Late gestation study

#### 2.4.1 Imaging protocol

21 healthy pregnant individuals with gestational age (GA) between 36 and 40 weeks were scanned at 0.55 T (Figure 2B). For each subject, HASTEs, bSSFPs and PC sequences of the thorax were acquired in several orientations. The low-field optimized bSSFP and PC sequences as described in Table 1 were used, where higher FAs and lower BWs were used, while maintaining image resolution similar to higher field strengths. The HASTE parameters were as follows: FOV = 450 × 450 mm^2^, in-plane resolution = 1.5 × 1.5 mm^2^, slice thickness = 4.5 mm, TR = 2,500 ms, TE = 106 ms, and FA = 180° (29).

Between 6 and 12 HASTE sequences were acquired for each subject covering the whole uterus and the fetal body. They were acquired in multiple orthogonal planes and reconstructed into an isotropic 3D volume with a resolution of 0.8 mm^3^ using dSVR (12). Compared to HASTE sequences acquired at higher field strengths, which typically have a resolution of 0.5 × 0.5 × 3 mm^3^, a lower resolution was used at 0.55 T (1.5 × 1.5 × 4.5 mm^3^) to compensate for the reduced SNR. Therefore, a higher number of input stacks is required for a reconstruction (at least six, whereas at 1.5 T or 3 T typically three good quality input scans is sufficient). Each reconstructed thorax was reoriented to a standard plane (39), and 10 vessels were segmented using a previously developed MONAI-based framework for automated multi-class fetal cardiac vessel segmentation using an Attention U-net and VoxelMorph (40–42). This network was originally trained on data acquired at 1.5 T, and was not re-trained for this study, thereby investigating the transferability of this network. The following vessels were segmented: Main Pulmonary Artery – MPA, Left Pulmonary Artery – LPA, Right Pulmonary Artery – RPA, Arterial Duct – AD, Ascending Aorta – AAo, Brachiocephalic Artery – BCA, Left Common Carotid Artery – LCCA, Left Subclavian Artery – LSA, Descending Aorta – DAo, and Superior Vena Cava – SVC.

#### 2.4.2 HASTE dSVR reconstruction and automatic vessels segmentation quality evaluation

Image quality evaluation of the dSVR reconstructions and automatic segmentations (similar to the bSSFP image assessment) were performed. For the HASTE dSVR reconstructions, the same scoring criteria as described in Section 2.2.2 was employed, with the reconstructed volume divided into six anatomic segments as described by Lloyd et al. (14): the systemic veins, the pulmonary veins, the pulmonary arteries, ductal arch, aortic arch, and the head and neck (H&N) vessels.

For the automatic segmentation of great vessels from the HASTE dSVR reconstruction, as there were no manual ground truth segmentations available, a visual evaluation was performed to assess the segmentation quality. For each vessel segmentation, a score of 0–3 was assigned. The scores had the following definitions: 0: segmentation failed, 1: segmentation present with major manual refinement required, 2: segmentation present with minor manual refinement required, 3: segmentation present with no/minimal manual refinement required. The average segmentation score for each structure was calculated.

#### 2.4.3 Blood flow measurement and ultrasound comparison

MRI flow measurements were calculated using retrospectively-gated PC sequences acquired in the late gestation cohort. The DAo, UV and SVC were acquired and measured as described in Sections 2.3. The optimized sequence parameters described in Table 1 were used. Mean flow was calculated as described in Section 2.3. To estimate the fetal weight from MRI, the fetal body was first automatically segmented from the bSSFP images using an in-house pre-trained U-net network based on the MONAI framework to measure the fetal volume (40, 43). The volume was then used to estimate the fetal weight using a formula described by Baker et al. (44).

7 Ultrasonographic measurements were performed by a single operator on various Voluson devices (Figure 2B). All measurements were performed within 48 h of MRI flow measurements. Umbilical venous flow was measured with 2D and color Doppler in a free loop. The umbilical vein diameter was measured by ultrasound at the site of Doppler measurement in the axial view. Estimated fetal weight was calculated from fetal measurements using the Hadlock 3 equation (45). Umbilical vein flow was calculated with the following equation:

(1) $$\begin{array}{rl} & \mathrm{Vessel}\;\mathrm{Flow}\;(\mathrm{mls}/\min)=\\ & \;\mathrm{Vessel}\;\mathrm{Area}\;(cm^{2})\times \mathrm{Mean}\;\mathrm{Velocity}\;(\mathrm{cm}/s\;)\times 60\;(s/\min) \end{array}$$

Mean velocity was calculated as half of the peak velocity, as per the established method (46, 47). Flow was normalized for estimated fetal weight. The UV flow measured with ultrasound in a free loop was compared with the UV as measured by MRI intra-abdominally with a Bland-Altmann plot in order to determine the agreement of the MRI flow measurements with the current clinical state of the art method.

## 3 Results

### 3.1 bSSFP sequence optimization

#### 3.1.1 Sequence optimization

**Figure 3:**
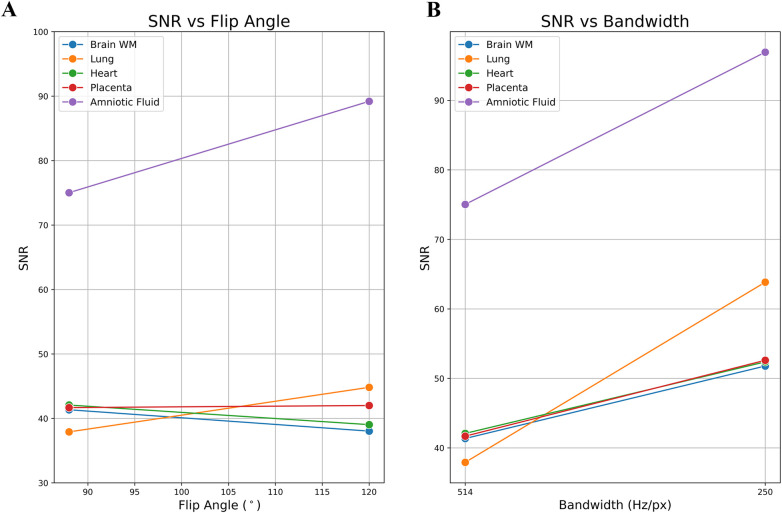
Example of a single subject's *in utero* SNR measurements: optimized parameters vs. original parameters. **(A)** SNR vs. flip angle. **(B)** SNR vs. bandwidth. The SNR of the lung and the amniotic fluid are most impacted by the flip angle.

**Figure 4:**
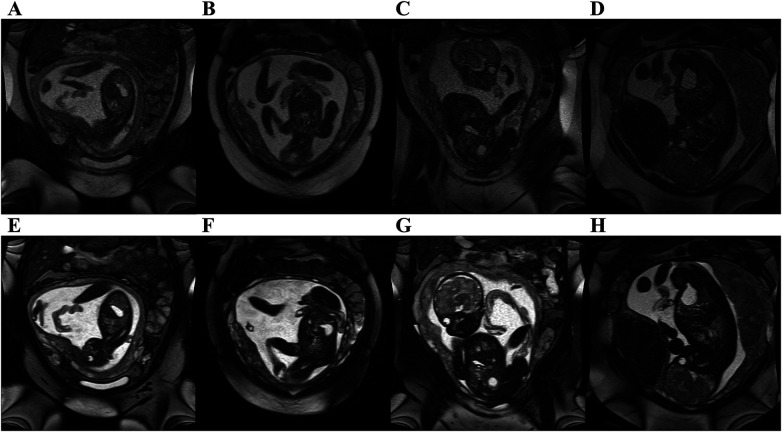
bSSFP fetal images acquired at 0.55 T with original 1.5 T parameters (top row) and optimized parameters at 0.55 T (bottom row). Original parameters: bandwidth = 514 Hz/Px, flip angle = 88°. Optimized parameters: Bandwidth = 250 Hz/Px, flip angle = 120°. All images had in plane resolution of 0.7 × 0.7 mm^2^. **(A, E)** GA: 25 weeks, **(B, F)** GA: 25 weeks, **(C, G)** GA: 30 weeks, **(D, H)** GA: 36 weeks. Note the improved SNR, and improved contrast between amniotic fluid and fetal structures.

A total of 14 subjects (GA: 18–38 weeks) were scanned between June 2023 to September 2023. Coronal stacks were acquired in 4/14 cases for *in utero* parameter optimization, the optimized parameters were then used to acquire bSSFP stacks in all three planes for the remaining 10 cases (Figure 2A). The optimized parameters showed a 1.6-fold SNR increase across four cases where images were acquired with both original and optimized parameters, which was similar to the result of the phantom experiments (Supplementary Material). Increasing the FA from 88° to 120° enhances SNR in the lung and amniotic fluid but slightly reduces it in brain white matter and heart (Figure 3A), where reducing the BW from 514 to 250 Hz/Px led to increased SNR in all anatomical regions (Figure 3B). The phantom experiments showed a 2.5-fold SNR difference between 1.5 T and 0.55 T (Supplementary Material), therefore, the 1.6-fold SNR increase with the optimal parameters compensates for approximately 65% of the SNR loss when transferring from 1.5 T to 0.55 T. Figure 4 shows the bSSFP images of the four fetuses used for sequence optimization, where images were compared between original and optimized parameters across a range of GAs (25–36 weeks). The optimized parameters notably enhanced image quality by increasing SNR and image contrast. Amniotic fluid signal significantly improved, thereby enhancing contrast. Fetal thorax SNR and contrast also improved without inducing artifacts. The optimized sequence resulted in a longer acquisition time of 42 s, compared to the original acquisition times of 25 s on 0.55 T (20 s on 1.5 T). Our study suggests images can have a slice thickness of 4.0 or 4.5 mm without compromising quality.

#### 3.1.2 bSSFP: image quality evaluation

**Figure 5:**
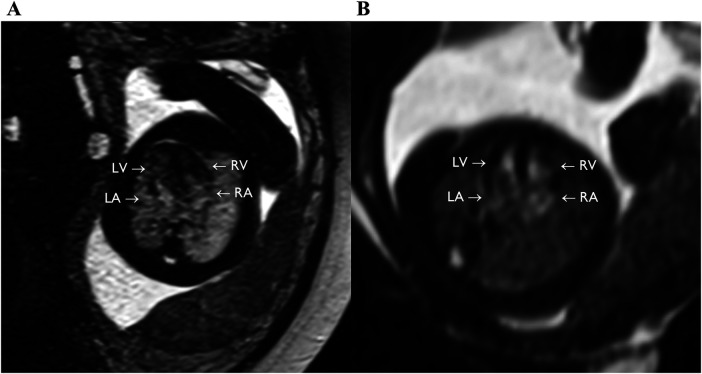
bSSFP images of the feal heart still showing equivalent to four chamber view (axial plane), with full visualization of the four chambers (LV, RV, LA, RA). **(A)** GA: 36 weeks. **(B)** GA: 24 weeks.

**Figure 6:**
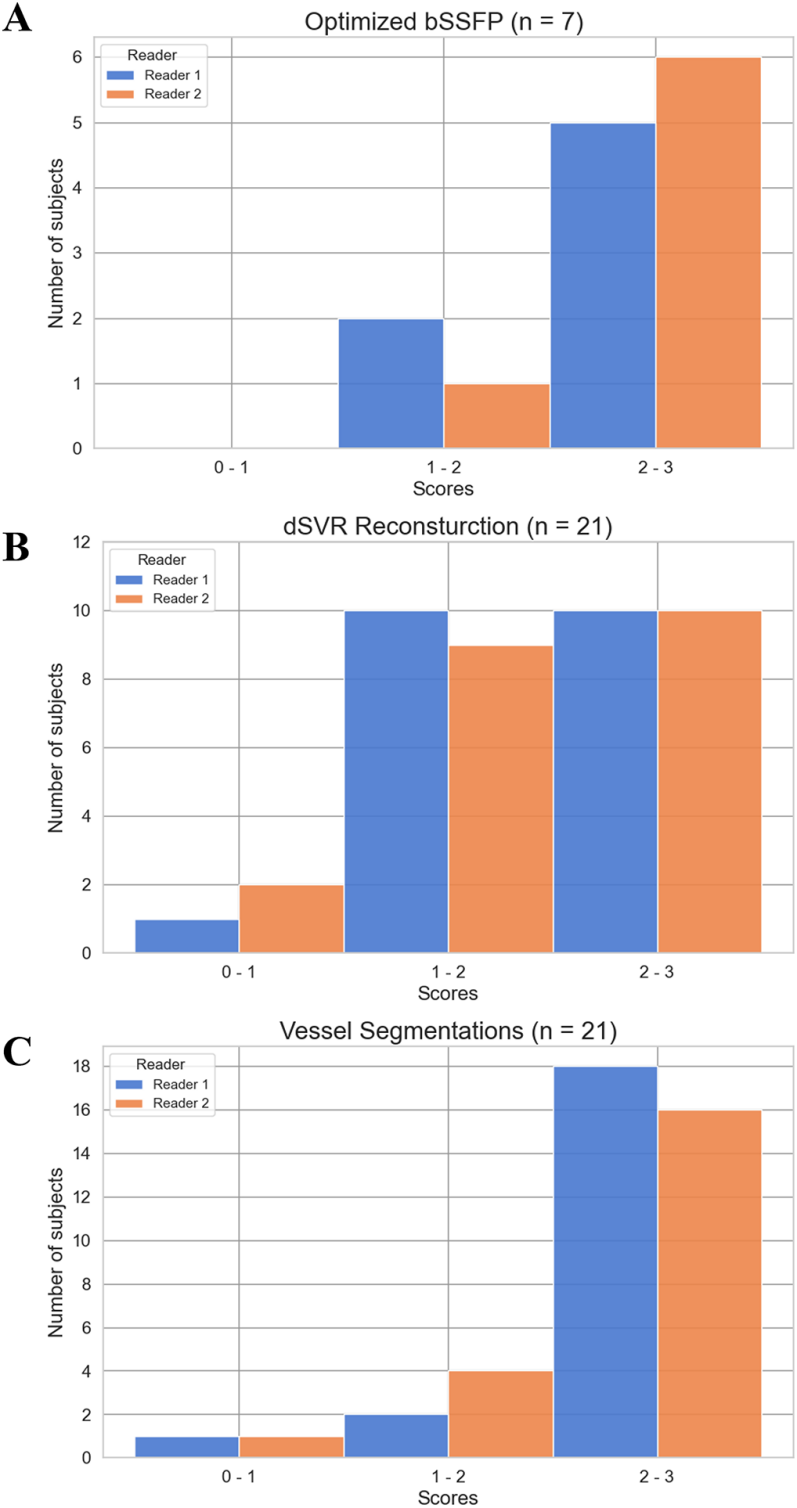
Score distribution of subjects for the image quality evaluations. The average score for each subject across all structures/regions assessed was calculated. **(A)** Optimized bSSFP sequence (*n* = 7). **(B)** dSVR reconstruction of HASTE stacks (*n* = 21). **(C)** Automatic vessel segmentations (*n* = 21).

A total of 10 subjects were acquired with the optimized bSSFP sequence in three orthogonal planes. Of the ten cases, three were excluded: one due to an early gestational age (18 weeks, too small to visualize structures), and two due to significant motion (GA: 30 and 38 weeks) (Figure 2A). Across seven remaining cases, the cardiac chambers (LA, RA, LV, RV) and systemic veins (SVC/IVC) were visualized in over 90% of cases, with the outflow tracts (LVOT/RVOT) and the aortic arch the most difficult structures to visualize consistently (Table 2, Figure 5). All subjects had an average score ≥ 1 and overall image quality was good with average scores of 2.06/3.00 and 2.08/3.00 (Figure 6A), visualization percentage was also comparable for the two readers (Table 2).

**Table 2:** Fetal cardiovascular structures and visualization assessment for seven cases with bSSFP sequences in three orientations with the optimized parameters.

Structure	Average score (*n* = 7)		Visualization (%)	
	Reader 1	Reader 2	Reader 1	Reader 2
Cardiac position	2.00 (0.45)	2.86 (0.35)	100	100
RV	2.00 (0.53)	2.71 (0.70)	86	86
LV	2.14 (0.53)	2.71 (0.70)	86	86
RA	2.14 (0.35)	2.71 (0.70)	100	86
LA	2.71 (0.35)	2.71 (0.70)	100	86
SVC	2.71 (0.45)	2.14 (0.64)	100	86
IVC	1.29 (0.45)	2.29 (0.70)	100	86
LVOT	1.86 (0.70)	1.14 (0.83)	43	43
RVOT	1.57 (0.64)	1.43 (0.49)	71	43
Aortic arch	1.86 (0.49)	1.00 (0.53)	57	14
Dao	1.71 (0.83)	1.71 (0.45)	57	71
Ductal arch	2.00 (0.70)	1.57 (0.49)	57	57
Average	2.06 (0.44)	2.08 (0.65)	80	70

For each structure, a score of 0 (not visible) to 3 (excellent visibility) was assigned. For the visualization assessment, a score of 0 and 1 were regarded as not visible, and scores of 2 and 3 were regarded as visible.

Values are mean (SD) or percentage.

### 3.2 Phase contrast sequence optimization and blood flow measurement

**Figure 7:**
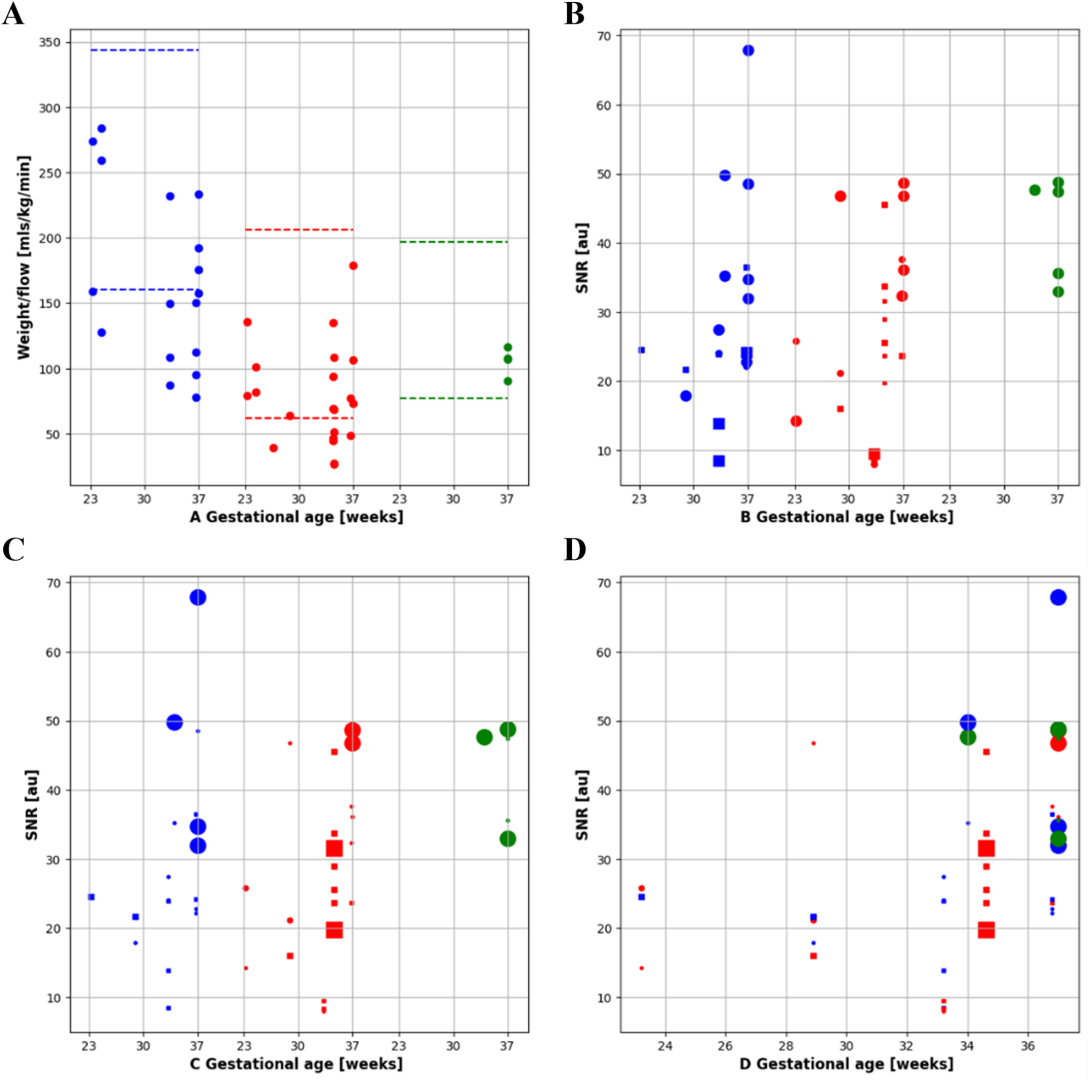
Quantitative flow and SNR results. **(A)** Indexed flow: flow indexed to fetal weight in descending aorta (DAo, blue), umbilical vein (UV, red) and superior vena cava (SVC, green) by gestational age. **(B-C)** SNR for DAo, UV and SVC, diamond = grappa 1, square = grappa 2 and dot = grappa 3. **(B)** Size encodes the flip angle and **(C)** size encodes the Bandwidth. **(D)** All results combined, size encoding bandwidth over gestational age.

**Figure 8:**
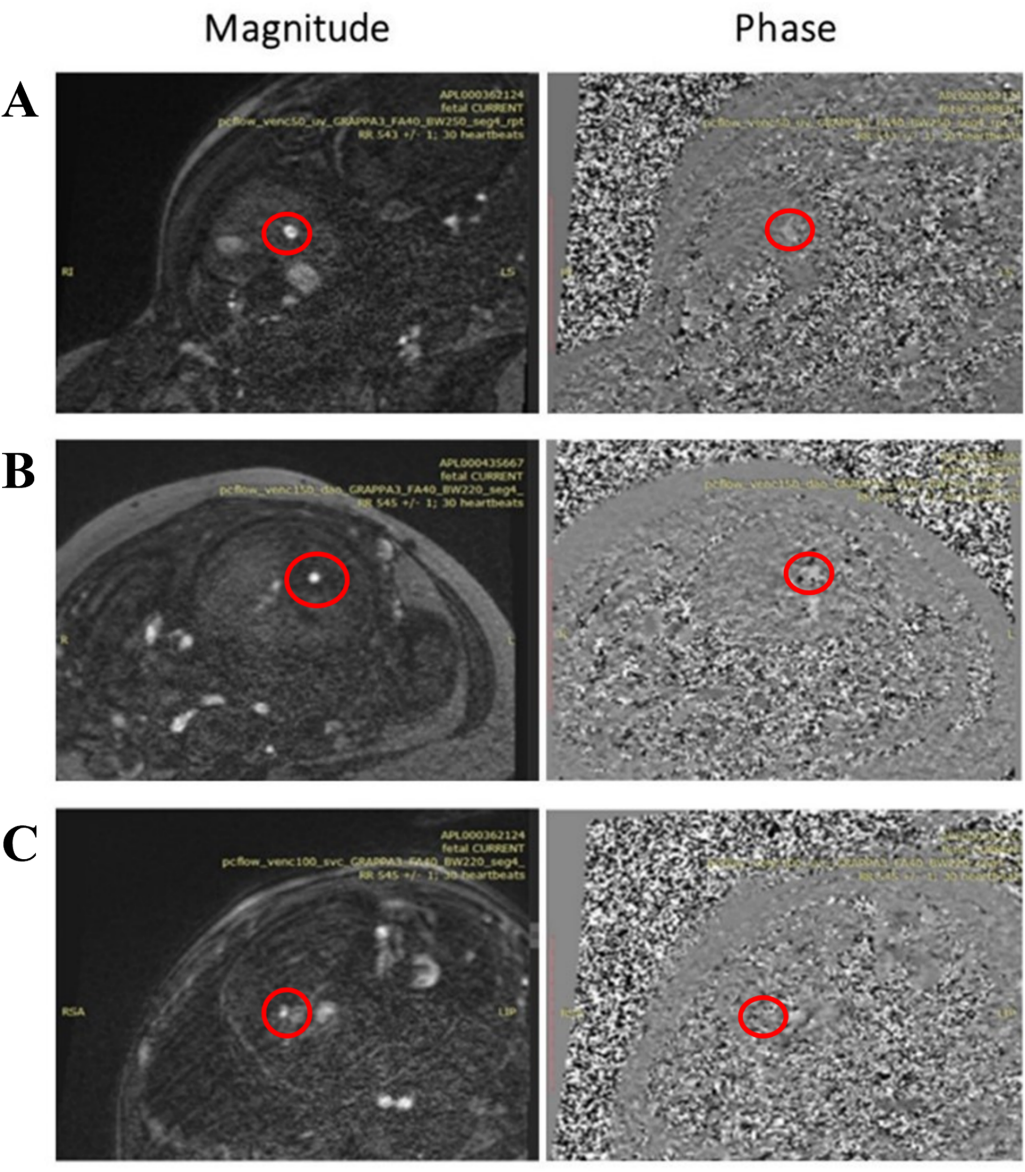
2d PC magnitude and phase images after metric optimized gating (MOG) acquired with 0.55 T scanner in a 37 weeks GA participant of **(A)** umbilical vein (UV), **(B)** descending aorta (DAo), and **(C)** superior vena Cava (SVC) with resolution 1.4 × 1.4 × 5 mm^3^, Acceleration Factor GRAPPA = 3, Flip Angle (FA) = 40°, Bandwidth (BW) = 250 Hz/Px for the UV and 220 Hz/Px for the DAo and SVC, and Segments = 4.

PC sequences were acquired in 9 subjects (GA: 23–37 weeks) between September and November 2023 for *in utero* parameter optimization (Figure 2A). The initial PC sequence parameters translated from 1.5 T parameters resulted in a scan time of 1 min when applied at 0.55 T and therefore showed visible motion artifacts. The optimal parameters were determined to be TR/TE = 78.64/5.31 ms, FOV = 300 × 300 mm^2^ resolution = 1.4 × 1.4 × 5.0 mm^3^, phase encoding lines = 69, FA = 40°, BW = 220 Hz/Px, GRAPPA = 3, and segments = 4 (Table 1), combination of TR = 98.3 ms, phase encoding lines = 104, GRAPPA = 2 and segments = 5 also showed similar improved image quality. The optimized sequence resulted in increased SNR and reduced motion artifacts in general, as well as a scan time of less than 20 s. (Figures 7, 8).

A total of 78 2D PC sequences were acquired across all subjects. After visual assessment, 42 sequences were of sufficient quality for metric optimized gating and flow analysis (UV: 21/45, DAo: 17/23, SVC: 4/10). The optimized sequence shows improved reconstruction quality using MOG, flow measurements for the UV, DAo, and SVC were successfully carried out despite the reduced SNR at 0.55 T. Minor motion artifacts were seen on the SVC vessel (Figure 8); however, blood flow was still measurable.

### 3.3 Late gestation study

#### 3.3.1 HASTE dSVR reconstruction and automatic vessels segmentation quality evaluation

19 late-gestation participants were scanned between November 2023 and February 2024, with 2 participants scanned twice for a total of 21 subjects (GA: 36–40 weeks). bSSFP and HASTE scans were acquired in all subjects, and PC scans were acquired in 15 participants, with one subject scanned twice and had PC sequences acquired in both scans, for a total of 16 subjects (Figure 2B).

For the HASTE dSVR reconstruction, the average visualization percentage across all structures was 66% and 64% across the two observers. The aortic arches (2.10/3.00, 1.90/3.00) and ductal arches (2.24/3.00, 2.14/3.00) were generally well visualized across all datasets. The pulmonary veins (1.95/3.00, 1.71/3.00) and pulmonary arteries (1.86/3.00, 1.81/3.00) had satisfactory visualization, and the head and neck vessels (1.52/3.00, 1.19/3.00) were most difficult to visualize consistently (Table 3). There were similar number of subjects with average scores of 1–2 and 2–3, and only 1–2 subjects with average scores of 0–1 (Figure 6B).

**Table 3:** Fetal cardiac vasculature assessment for 21 HASTE dSVR reconstructions.

Anatomic segments	Average score (*n* = 21)		Visualization (%)	
	Reader 1	Reader 2	Reader 1	Reader 2
Systemic veins	1.52 (0.50)	1.57 (0.50)	52	57
Pulmonary veins	1.95 (0.65)	1.71 (0.55)	76	67
Pulmonary arteries	1.86 (0.77)	1.81 (0.73)	62	62
Ductal arch	2.24 (0.75)	2.14 (0.64)	81	86
Aortic arch	2.10 (0.75)	1.90 (0.53)	76	81
Head and neck	1.52 (0.59)	1.19 (0.66)	57	33
Average	1.84 (0.27)	1.69 (0.30)	66	64

Six anatomic segments were visually inspected. For each structure, a score of 0 (not visible) to 3 (excellent visibility) was assigned, and the average across all 21 structures was taken. For the visualization assessment, a score of 0 and 1 were regarded as not visible, and scores of 2 and 3 were regarded as visible.

Values are mean (SD) or percentage.

**Figure 9:**
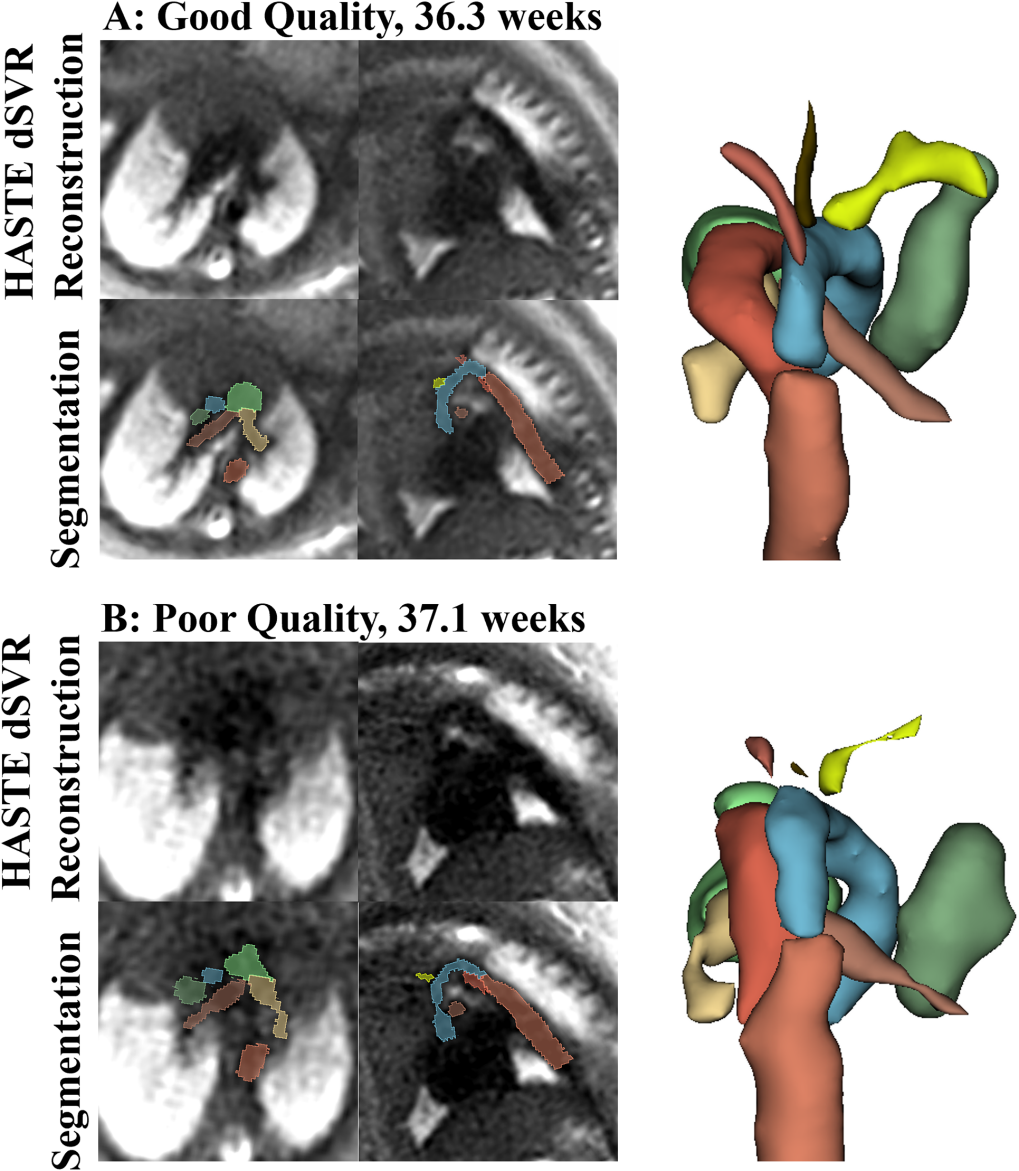
Examples of good quality **(A)** and poor quality **(B)** HASTE dSVR reconstructions and vessel segmentations from the HASTE reconstructions, in the axial and sagittal fetal view. **(A)** Reconstruction average score across structures: 2.50, as the IVC was partially visualized; Segmentation Score across structures: 3.00; **(B)** Reconstruction average score across structures: 1.17, due to poor vessel separation; segmentation score across structures: 1.16.

The automatic segmentation of the great vessels performed on the HASTE dSVR reconstructions were mostly successful and showed good fitting compared to the ground truth based on visual inspection. The ten vessels segmented had an average quality score of 2.35/3.0 for both readers (Table 4), indicating the segmentations on average only required minimal refinements. Among the vessels segmented, the AD had the highest average quality score between the two readers (2.74/3.00) while the AAo and BCA had the lowest (1.96/3.00 and 1.77/3.00). Most subjects had good overall segmentations with average scores across all vessels ≥ 2 (16/21 and 18/21) with one subject where the segmentations were poor and had average scores ≤ 1 (Figure 6C). Examples of good and poor quality reconstructions and segmentations can be seen in Figure 9.

**Table 4:** Quality assessment of the automatic segmentation of fetal cardiac great vessels of 21 cases.

Vessels	Average score (*n* = 21)	
	Reader 1	Reader 2
MPA	2.76 (0.75)	2.67 (0.71)
LPA	2.57 (0.81)	2.67 (0.78)
RPA	2.48 (0.81)	2.38 (0.90)
AD	2.71 (0.72)	2.76 (0.75)
AAo	1.86 (0.91)	2.05 (0.84)
BCA	1.86 (1.11)	1.67 (0.89)
LCCA	2.19 (0.98)	1.90 (1.06)
LSA	2.10 (1.04)	2.29 (0.98)
DAo	2.71 (0.56)	2.71 (0.55)
SVC	2.48 (0.81)	2.76 (0.61)
Average	2.35 (0.33)	2.35 (0.38)

Ten vessels were automatically segmented. For each vessel, a score of 0 (segmentation failed) to 3 (excellent segmentation with no/minimal refinement required) was assigned, and the average value of each vessel was calculated.

Values are mean (SD).

#### 3.3.2 Blood flow measurement and ultrasound comparison

**Figure 10:**
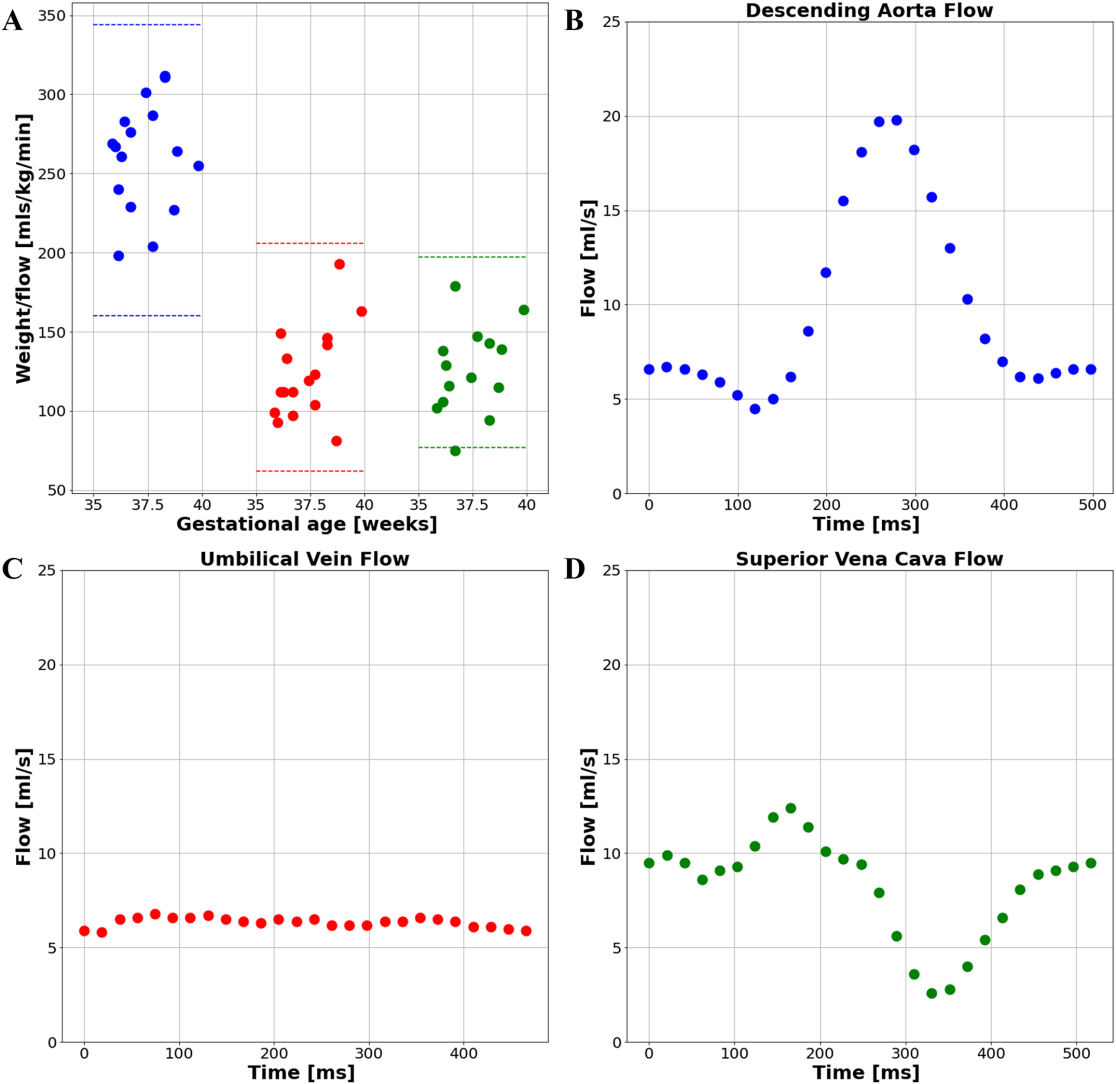
**(A)** flow measurements from the late gestation study cohort. Blue: DAo; Red: UV; Green: SVC; Example subject's flow curve from **(B)** the descending aorta, **(C)** the umbilical vein, and **(D)** the superior vena cava.

The UV, DAo and SVC were acquired for each subject. A total of 103 PC sequences were acquired and 95 were of sufficient quality for further analysis (UV: 34/37, DAo: 34/36, SVC: 27/30). Flow with respect to gestational age can be seen in Figure 10A. No trend with gestational age was seen. If multiple sequences per vessel were acquired, the highest flow measurement was taken. All flow measurements were within the expected range per vessel, indicated by the dotted lines. Example flows over time from each vessel can be seen in Figures 10B–D.

**Figure 11:**
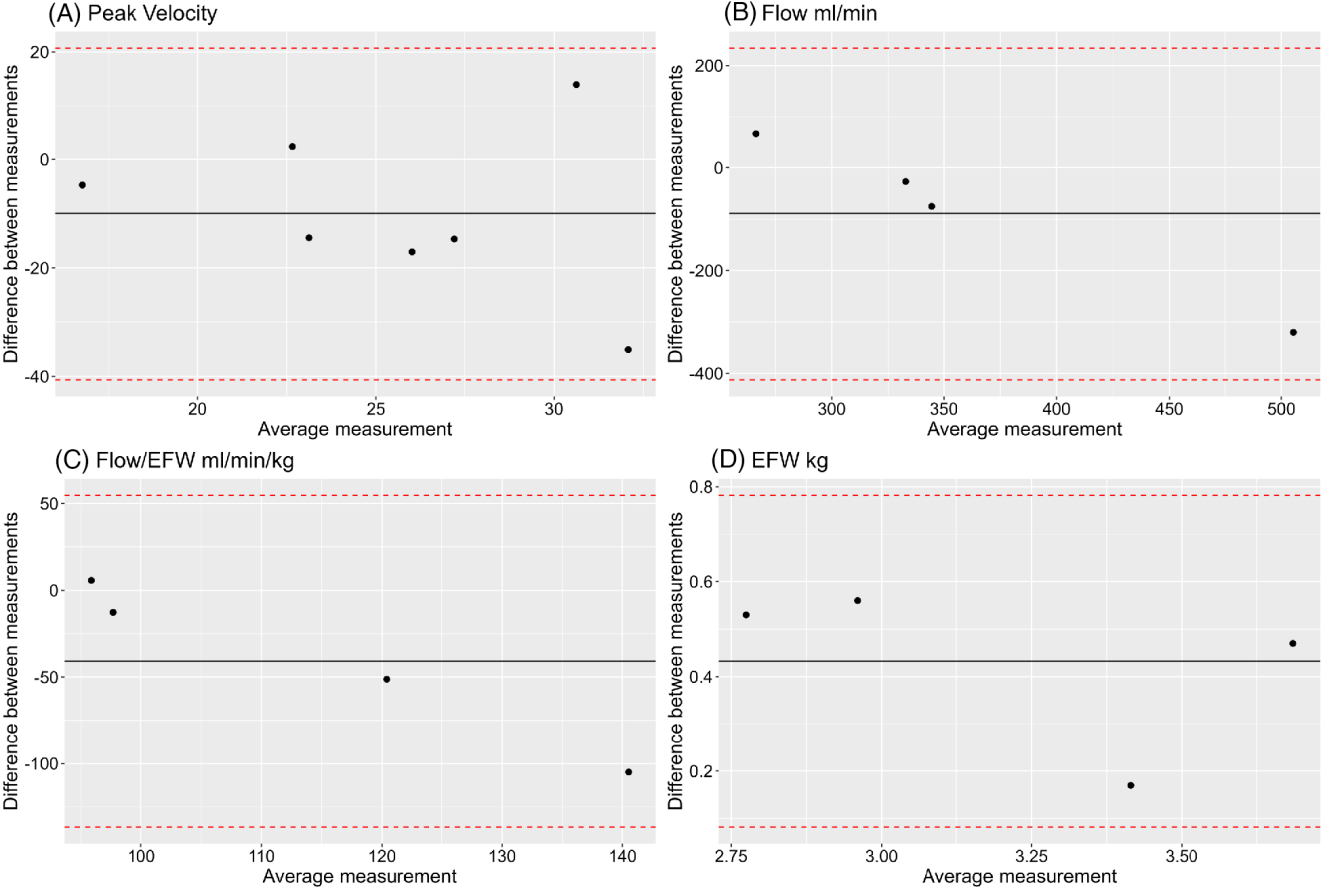
Bland Altman plots showing comparison between MR and US measurements of **(A)** umbilical vein peak velocity, **(B)** umbilical vein flow, **(C)** umbilical vein flow adjusted for estimated fetal weight, **(D)** estimated fetal weight (EFW).

Ultrasound measurement of peak velocities was obtained in 7 cases with matched MRI measurement (Figure 2B). Peak velocity measurements were compared with a Bland Altman Plot (Figure 11A). This plot may suggest that the difference between MR and US venous Doppler is larger at higher flows. Out of the 7 cases, 3 of them had no umbilical vein diameter measurement available, Bland Altman plots for comparing umbilical venous flow and adjusted flow in the remaining 4 cases appear not to correlate well when measured by MR and US (Figures 11B,C). Estimated fetal weight correlates well between US and MRI for these cases (Figure 11D).

## 4 Discussion

### 4.1 bSSFP signal-to-noise ratio optimization

The optimized bSSFP sequence at 0.55 T demonstrated increased SNR and improved image quality through parameter optimization. Notably, lower bandwidths and higher flip angles enhanced SNR, consistent with previous findings (30). This demonstrates the dynamic relationship between different sequence parameters and the SNR, highlighting the importance of sequence optimization at low field strengths to increase the SNR, as well as the benefit of the lower SAR at low field strengths. The 1.6-fold increase in SNR after parameter optimization effectively compensates for 65% of the overall SNR loss when moving from 1.5 to 0.55 T. The investigation into the SNR across different anatomical regions demonstrated that a reduction in bandwidth corresponds to a uniform increase in SNR for all regions. Meanwhile, increasing the flip angle results in greater disparity in SNR levels among the various regions, indicating improved contrast with the higher flip angle. In addition to increased contrast from the higher flip angle, the bSSFP sequence's inherent contrast, tied to the T2/T1 ratio, further enhances contrast due to the increased T2/T1 ratio at low field. This highlights the advantage of using the bSSFP sequence with optimal parameters at low field for fetal cardiac examinations. Our observations indicate that a slightly thinner slice thickness compared to the original setting does not degrade image quality, this allows our sequence to achieve a resolution comparable to standard clinical protocols on 1.5 T, showcasing the potential of low-field fetal cardiac applications. The acquisition time is extended compared to 1.5 T, increasing from 20 s to 42 s. However, we observed no significant motion artifacts induced by the longer acquisition, especially in late gestion. The image quality evaluation showed good visualization of fetal cardiac structures on the optimized sequence, demonstrating its potential as a diagnostic tool.

### 4.2 Phase contrast sequence optimization and blood flow measurement

We showed that by optimizing the PC sequence parameters, we can acquire data of sufficient quality for subsequent retrospective gating and blood flow measurements. The optimized bSSFP sequence also played a key role in the planning of PC sequences. With higher quality bSSFP images, effective planning can be carried out to locate the target great vessels. During the optimization, the number of PC sequences acquired with sufficient quality for further analysis was low (42/78), this was mainly due to challenges at the study's onset, such as the process of optimizing sequence parameters and imaging protocols. Moreover, subjects at an earlier GA were also acquired, increasing the fail rate of the acquisition. In the cases where the acquired images were of sufficient quality, retrospective gating was successful using MOG, subsequent blood flow measurements of the UV, DAo, and SVC were carried out successfully with values falling within the expected range (48). Although using a Doppler ultrasound device for real time gating and blood flow measurements has been shown to be effective for fetal blood flow measurements (18), previous work has shown that gating using MOG and a Doppler ultrasound device for blood flow measurements obtained comparable results (49) and has the advantage of not requiring an additional device, although an extra software tool is required. The successful blood flow measurement at 0.55 T demonstrates the broader application and diagnostic potential of low field fetal functional cardiac imaging.

### 4.3 Late gestation study

The clinical study on late gestation fetuses showed the potential of using 0.55 T MRI as a diagnostic tool for CHD. The commonly used HASTE sequence combined with 3D reconstruction using dSVR at 1.5 or 3.0 T to examine the fetal cardiac vasculature were adapted to 0.55 T. Whilst the reconstruction quality had a visualization percentage of 60% across all fetal cardiac structures under test, certain structures (the aortic and ductal arches and pulmonary veins) had a more consistent visualization. In a similar study conducted on 1.5 T (14), the dSVR reconstruction showed better visualization of fetal cardiac structures (>90%) and higher image scores for individual anatomic segments. However, In the current study, a relatively low number of input stacks was used - increasing the number of HASTE stacks acquired and optimizing for SVR may improve visualization at 0.55 T in the future. Although trained on a network that was trained using data acquired at 1.5 T, subsequent automatic segmentation of the great vessels showed excellent generalizability and performance when utilized with 0.55 T data, where 18/21 cases required no or minor refinements upon visual inspection (average score > 2). Finally, most of the PC sequences acquired were of sufficient quality for analysis (95/103), demonstrating the reliability of the optimized PC sequence when applied on late gestation subjects, subsequent flow measurement after retrospective gating was successful in all subjects. All flow values were within the expected range, and the shape of the flow curves for different vessels matches previous work done on higher field strengths (50). Our results demonstrate the feasibility of a fetal cardiac examination protocol at 0.55 T, the adapted and optimized sequences have the potential to be used clinically for a comprehensive morphological and functional analysis of the fetal cardiovascular system.

In the ultrasound-MRI UV comparison, the number of cases we present is low, therefore it is difficult to fully interpret the data. However, based on the Bland-Altmann plots of the small number of data points, there appears to be agreement as all points lie within ±1.96 standard deviations of each other. Differences in flow on MR and ultrasound can be accounted for by location of measurement, observer variability, and differences in fetal weight estimation calculations between the two modalities. The MR established protocol for measuring flow in the umbilical vein is at its abdominal portion, while ultrasound protocols measure flow in a free loop or intra-abdominally. Whilst umbilical vein elasticity, diameter, and peak velocity vary across the length of the cord (51, 52) it has been shown that average flow does not differ (53). Further studies are required to see if this difference is significant when flow measurements derived from both modalities. Fetal estimation weights by MR and ultrasound have been established to correlate well, with MR performing better when compared with actual birth weight (46). Differences in adjusted flows can be explained by the inherent differences in calculating fetal weight the imaging modalities. For ultrasound, weight is calculated from head circumference, abdominal circumference, and femur length. These measurements don't account for additional soft tissue mass towards later gestation, making this method of weight estimation less reliable. MR weight is calculated from body volume following segmentation. A larger sample size is needed to determine if these two modalities are comparable for measuring umbilical vein flows.

### 4.4 Limitations & future work

Our study demonstrated the improved SNR of the optimized bSSFP and PC sequences. However, due to the spatially varying noise amplification introduced in parallel imaging, our method can only measure the apparent SNR, which may introduce errors (54). Future work could apply methods suitable to parallel imaging for more rigorous SNR quantification (55, 56). Our results at 0.55 T are encouraging, however, a direct comparison of the optimized sequences at 0.55 T to the optimized sequences at 1.5 T or 3.0 T with the same subjects is lacking. Future work should investigate direct comparisons of SNR, flow quantification, and blinded image scoring, for both healthy and CHD subjects. Such comparisons would help investigate the practical value of low-field-strength scanning in diagnosing CHD, and provide insights into the useability of 0.55 T fetal cardiac MR compared to the useability of 1.5 T. For cardiac gating, we only investigated retrospective gating using MOG, future work could explore alternative gating methods such as direct gating using a Doppler ultrasound device. Our study was done entirely on healthy subjects, and the diagnostic ability of using low field MRI for CHD patients requires further investigation. Our clinical analysis was limited to late gestation subjects (GA > 36 weeks), future work could be expanded to subjects with lower GAs. Our feasibility study used a single scanner at a single site and should be tested at other institutions and on different low field scanners. Lastly, we only explored static structural imaging, more comprehensive 3D visualization and exploration of fetal cardiac anatomy could be achieved by integrating 3D reconstruction with cardiac synchronization techniques to produce 4D cine images (8).

## 5 Conclusion

The lower cost and the larger bore size of the 0.55 T MRI system widens the accessibility of fetal cardiac MRI, and potentially aids the diagnosis and treatment planning of congenital heart diseases. In this study we demonstrate the feasibility of using a low-field strength (0.55 T) commercially available MRI scanner for comprehensive structural and functional fetal cardiac imaging. Strategically optimizing sequence parameters based on the properties of the lower field strength can effectively compensate for the reduced SNR at low field strength. The optimized bSSFP and PC sequence showed improved SNR and image quality, with subsequent blood flow measurements within physiological limits and comparable to contemporaneous ultrasound measurements. Finally, our late gestation study results demonstrate the potential of low field strength imaging for future diagnostic usage.

## Data Availability

The data is available from the corresponding author upon reasonable academic request (REC 441 21/LO/0742).
