# Supplementary material for: Structural and functional fetal cardiac imaging using low field (0.55 T) MRI
Source: Front Pediatr. 2024 Sep 3;12:1418645. doi: 10.3389/fped.2024.1418645 (PMC11421172; doi:10.3389/fped.2024.1418645)
Supplement: Supplementary file 1 [file Datasheet1.pdf]

Supplementary Material

1 bSSFP: Phantom Experiments

1.1 Methods

All scans were performed using a clinical 0.55T scanner (MAGNETOM Free.Max, Siemens Healthcare, Erlangen, Germany), using a 9-element spine coil integrated into the patient table alongside a 6-element flexible coil (BioMatrix Contour Coil, Siemens Healthcare, Erlangen, Germany) and a clinical 1.5T scanner (MAGNETOM, sola, Siemens Healthcare, Erlangen, Germany). A doped water-filled cylindrical phantom was used for all experiments and no shimming was applied. In order to provide a baseline for the bSSFP image quality, the current clinical bSSFP scanning parameters on a 1.5T scanner (TABLE 1) were used to scan the same phantom on 1.5T and 0.55T. The bSSFP sequence optimization on 0.55T focused on optimizing the flip angle (FA) and bandwidth (BW) of the sequence, the two parameters that heavily contribute to the SAR and image artifacts that are unable to be more optimal at 1.5T due to SAR limitations. The evaluated parameters can be found in Supp. TABLE 1. SNR was measured as the apparent SNR defined as the mean signal in a region of interest (ROI) divided by the standard deviation of a background region. For the SNR measurements, a spherical region with a volume of 500 mm^3 was selected at the center and background of the image, enabling separate measurements of signal and noise for the subsequent SNR calculation.

1.2 Results

The 1.5 vs 0.55T phantom experiments showed the expected 2.5-fold SNR difference across different in-plane resolution and slice thicknesses with the same BW and FA (Supp. Figure 1A and 1B). The bSSFP sequence parameter optimization showed that SNR is increased at lower BW of 250 Hz/Px over all Fas and slice thicknesses (Supp. Figure 1C). The SNR fluctuates slightly as FA increases and peaks at 120° (Supp. Figure 1D). A 1.6-fold average SNR increase was observed using the optimized parameters (TR/TE = 649.2/4.09 ms, BW = 250 Hz/Px, FA = 120°) when compared to the original 0.55T signal.

Supplementary TABLE 1. Original 1.5T bSSFP sequence parameters, parameters used in phantom experiments, and optimized parameters.

Sequence	TR (ms)	TE (ms)	BW (Hz/px)	FA (°)	FOV (mm^2)	In-plane Resolution (mm^2)	Slice Thickness (mm)	GRAPPA	Acquisition time (s)
bSSFP (1.5T)	569.4	3.11	514	88	350 x 350	0.7 x 0.7	5.0	2	25
bSSFP (Phantom)	476.2 – 697.7	3.11	250, 350, 514	60 - 150	350 x 350	0.7 x 0.7, 1.0 x 1.0, 1.25 x 1.25	3.5, 4.0, 4.5, 5.0	2	25 - 50
bSSFP (Optimized)	649.2	4.09	250	120	350 x 350	0.7 x 0.7	4.0 or 4.5	2	42

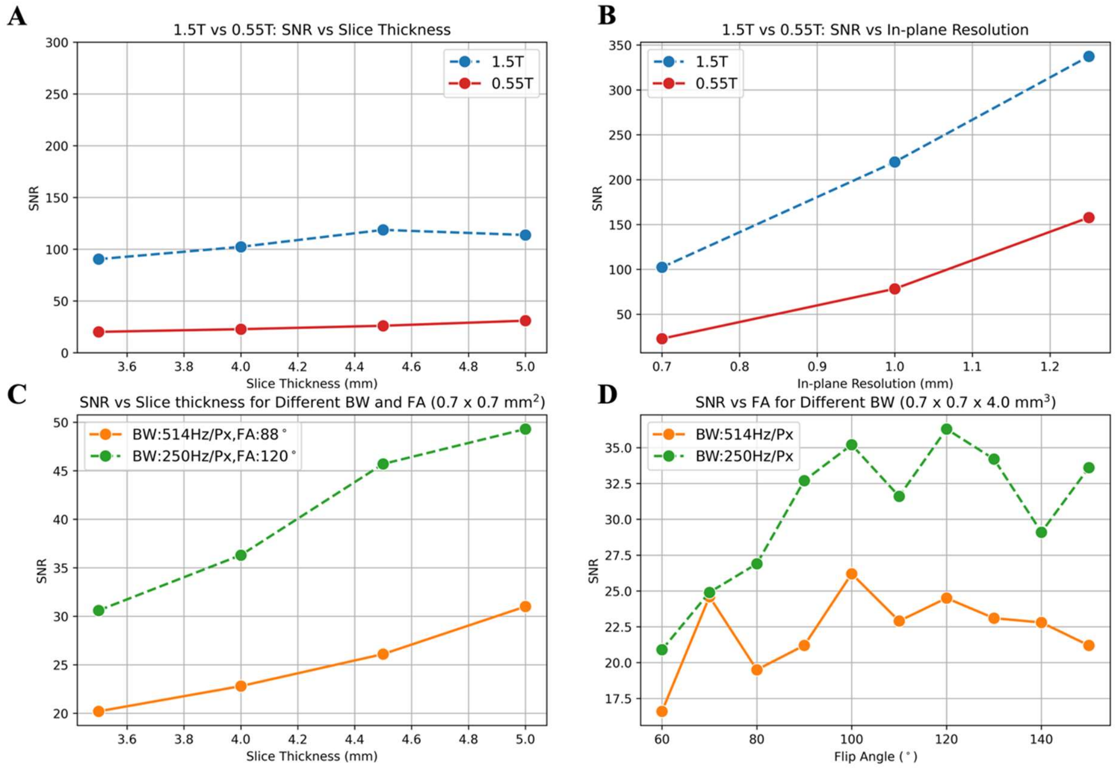

Supplementary Figure 1. Phantom experiment: bSSFP SNR comparison between 1.5T and 0.55T, and SNR optimization at 0.55T. (A) SNR vs slice thickness and (B) SNR vs in-plane resolution. (C) SNR vs slice thickness for different bandwidth and flip angle, with in-plane resolution of 0.7 x 0.7 mm^2. (D) SNR vs flip angle for different bandwidths with resolution of 0.7 x 0.7 x 4.0 mm^3. Overall, lower bandwidth at 250 Hz/Px and higher flip angle at 120° resulted in a 1.6-fold SNR increase compared to original parameters.
